# J-waves in acute COVID-19: A novel disease characteristic and predictor of mortality?

**DOI:** 10.1371/journal.pone.0257982

**Published:** 2021-10-14

**Authors:** Naufal Shamilevich Zagidullin, Lukas J. Motloch, Timur Ilgamovich Musin, Zilya Adibovna Bagmanova, Irina Alexandrovna Lakman, Anton Viktorovich Tyurin, Ruslan Mansurovich Gumerov, Dinar Enikeev, Benzhi Cai, Diana Firdavisovna Gareeva, Paruir Artakovich Davtyan, Damir Aidarovich Gareev, Halima Malikovna Talipova, Marat Rifkatovich Badykov, Peter Jirak, Kristen Kopp, Uta C. Hoppe, Rudin Pistulli, Valentin Nikolaevich Pavlov

**Affiliations:** 1 Department of Internal Medicine I, Bashkir State Medical University, Ufa, Russian Federation; 2 Department of Biomedical Engineering of Ufa State Aviation Technical University, Ufa, Russian Federation; 3 Clinic II for Internal Medicine, University Hospital Salzburg, Paracelsus Medical University, Salzburg, Austria; 4 Department of Economics, Finance and Business, Bashkir State University, Ufa, Russian Federation; 5 Department of Internal Diseases II, Bashkir State Medical University, Ufa, Russian Federation; 6 Department of Pharmacy at The Second Affiliated Hospital, and Department of Pharmacology (The Key Laboratory of Cardiovascular Medicine Research, Ministry of Education) at College of Pharmacy, Harbin Medical University, Harbin, China; 7 Department of Surgery, Bashkir State Medical University, Ufa, Russian Federation; 8 Department of Cardiology I, Coronary and Peripheral Vascular Disease, Heart Failure, University Hospital Münster, Münster, Germany; 9 Department of Urology, Bashkir State Medical University, Ufa, Russian Federation; Universitatsklinikum Wurzburg, GERMANY

## Abstract

**Background:**

J-waves represent a common finding in routine ECGs (5–6%) and are closely linked to ventricular tachycardias. While arrhythmias and non-specific ECG alterations are a frequent finding in COVID-19, an analysis of J-wave incidence in acute COVID-19 is lacking.

**Methods:**

A total of 386 patients consecutively, hospitalized due to acute COVID-19 pneumonia were included in this retrospective analysis. Admission ECGs were analyzed, screened for J-waves and correlated to clinical characteristics and 28-day mortality.

**Results:**

J-waves were present in 12.2% of patients. Factors associated with the presence of J-waves were old age, female sex, a history of stroke and/or heart failure, high CRP levels as well as a high BMI. Mortality rates were significantly higher in patients with J-waves in the admission ECG compared to the non-J-wave cohort (J-wave: 14.9% vs. non-J-wave 3.8%, p = 0.001). After adjusting for confounders using a multivariable cox regression model, the incidence of J-waves was an independent predictor of mortality at 28-days (OR 2.76 95% CI: 1.15–6.63; p = 0.023). J-waves disappeared or declined in 36.4% of COVID-19 survivors with available ECGs for 6–8 months follow-up.

**Conclusion:**

J-waves are frequently and often transiently found in the admission ECG of patients hospitalized with acute COVID-19. Furthermore, they seem to be an independent predictor of 28-day mortality.

## Background

The novel coronavirus disease COVID-19 was officially declared a pandemic on March 11, 2020 by the World Health organization. While the virus is still spreading rapidly among the human population, it has led to a worldwide health care crisis with over 195 million confirmed cases and over 4.1 million confirmed fatalities (as of July 30, 2021, WHO, https://covid19.who.int).

SARS-CoV-2 primarily affects the respiratory system, leading potentially to severe pneumonia and acute respiratory distress syndrome resulting in high mortality rates [1]. SARS-COV-2 enters its host cell by binding to the angiotensin converting enzyme 2 (ACE-2) receptor, followed by internalization [2, 3]. Given the broad expression of the ACE-2-receptor in multiple different cell types, virtually all organ systems can be directly affected by COVID-19, including the heart [4, 5]. Several studies have described histological changes of cardiac tissue in patients with COVID-19 indicative of a direct cardiac involvement [6, 7]. Similarly, cardiac MRI-studies revealed cardiac changes in COVID-19 survivors, suggestive of COVID-19-associated cardiac disease [8]. Accordingly, cardiac injury has been reported in 20–28% of hospitalized COVID-19 patients. This finding was also associated with a significant increase in mortality [9, 10]. In line with these observations, non-specific ECG alterations along with cardiac arrhythmias were reported in up to 16% of COVID-19 patients [11] and were associated with elevated troponin levels, thereby suggesting cardiac injury [11]. Furthermore, a study on critically ill COVID-19 patients reported ECG abnormalities occurring in 93% of this patient population, indicating an association of ECG changes with disease severity [12] with atrial fibrillation reported as the most frequent arrhythmia [13].

Given the association of disease severity and cardiac injury, ECG alterations might have a prognostic value in COVID-19 disease. Considering the broad availability, the low costs and the short amount of time needed for conducting an ECG analysis, a prognostic impact of ECG alterations would be of utmost value especially for health care systems severely affected by the pandemic with shortages in ICU beds and medical staff [14, 15]. Although ECG analysis can be realistically utilized even in overstrained medical systems, ECG patterns offering a potential prognostic value have yet to be identified. Ozdemir et al. have proposed a novel approach to the classification of COVID-19 ECG by using a hexaxial feature mapping along with deep learning [16]. In their study, they were able to achieve COVID-19 outcome prediction with an accuracy of 93.0% with emphasis on the impact of COVID-19 on ECG changes [16]. In addition, the analysis of J-waves might represent a promising approach in the context of COVID-19-induced ECG changes. Prominent J-waves presenting as late positive waves following the QRS complex are a common phenomenon reported in 5–6% of the general population [17, 18]. J-waves have also been observed in the context of hypothermia and hypercalcemia [19]. Moreover, J-waves are predictive of life-threatening arrhythmic events and sudden cardiac death, as well as cardiac ischemia [17, 19]. Interestingly, J- waves were also reported as occurring in COVID-19 disease [20, 21]. The authors of this manuscript also observed J-waves as a frequent finding during routine care of their COVID-19 patients. J-waves were most frequently encountered in 12-lead ECGs at the admittance of COVID-19 patients to the emergency department. Contrary, J-waves tended to dissolve during the ongoing course of the disease. Accordingly, the authors assumed that J-waves might predominantly occur at an early stage of COVID-19 disease and thus might also be of prognostic impact. To further elucidate their initial observations, the authors started screening for J-waves at patient admission in the emergency department. Similar, follow-up ECGs were conducted to further analyse the development of J-waves during the course of the disease. To the best of our knowledge, specific analyses of J-waves including their incidence and prognostic implications in COVID-19 have not yet been conducted, which is the topic of the present study.

## Materials and methods

### Study cohort, data collection and analyses

The study was performed in accordance with the standards of good clinical practice and the principles of the Declaration of Helsinki, receiving approval by the ethics commission of the Bashkir State Medical University (N11, 2020).

**Fig 1:**
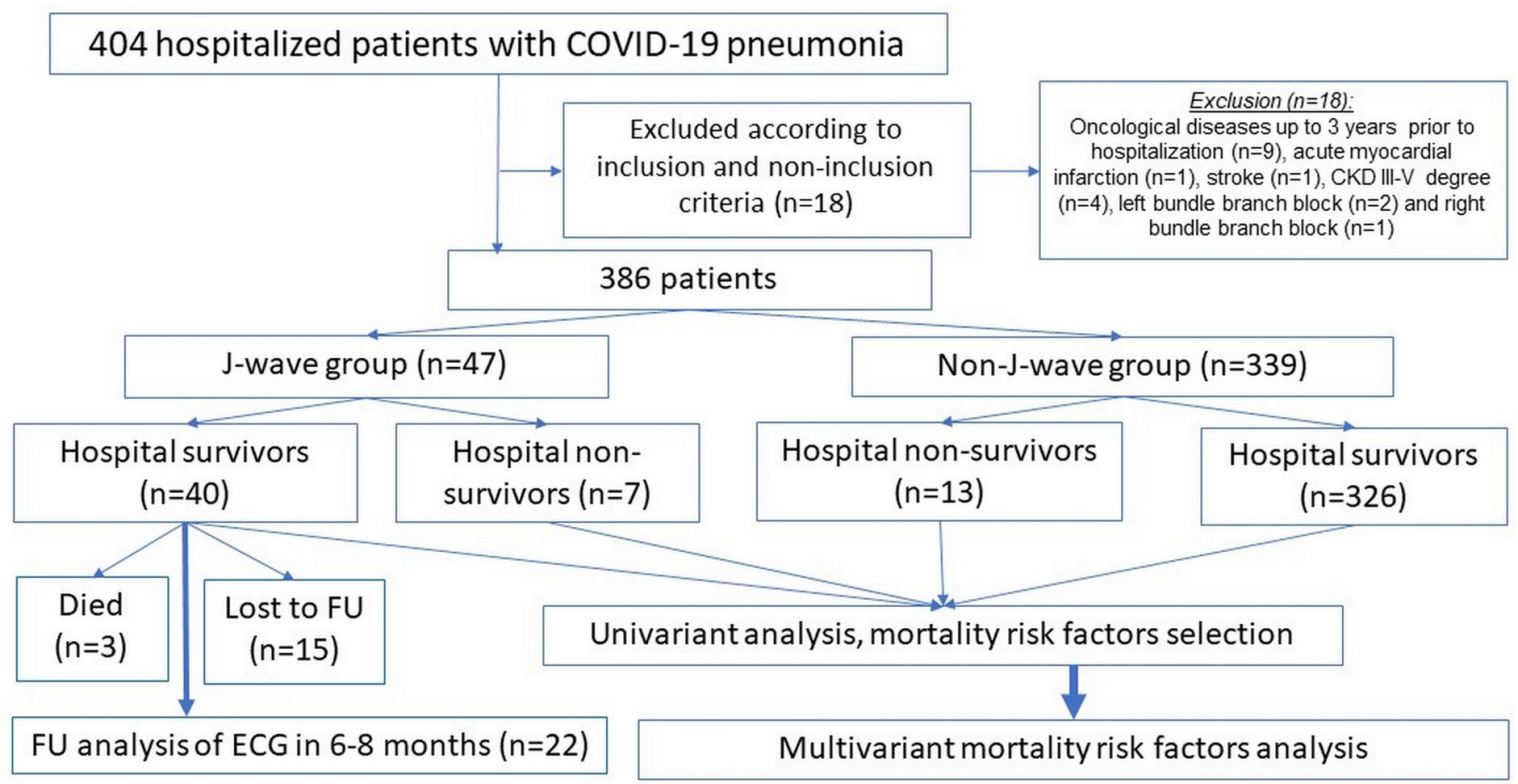
Study design.

In this single-center, retrospective study, 404 consecutive patients were screened, of whom 386 met inclusion criteria and none of the exclusion criteria and were therefore included in the study. All patients were hospitalized due to COVID-19 disease (Bashkir State Medical University Hospital, Bashkir State, Russian Federation) between May 1, 2020 and July 31, 2020 (Fig 1). All patients were 18 years or older and suffered from COVID-19-related pneumonia. Patients displaying potential confounder for ECG changes were not included in the study. Exclusion criteria included electrolyte disturbances, chronic kidney disease stage IV-V, malignant disease within the past three years, myocardial infarction, acute stroke, immunodeficient conditions and chronic heart failure NYHA III-IV. Also excluded were patients with preexisting ECG changes limiting the analysis of J-waves, such as higher degree AV-Block (type II and III), as well as left and right bundle branch block. In patients presenting with clinical characteristics suspicious for acute coronary syndrome, acute myocardial injury was ruled out by evaluating repetitive Troponin-I levels according to current ESC Guidelines [22]. Additionally, TTEs were evaluated to exclude patients with pericardial effusion and regional wall motion abnormalities [22, 23]. Patients with suspected acute stroke meeting the definition of current guideline definitions were not included [24, 25].

Medical history and clinical findings were captured. Blinded analysis of ECGs was conducted by three cardiologists with electrophysiological expertise. According to current consensus reports, presence of J-waves was diagnosed, if the peak of an end QRS notch (defined as notched J-wave) and/or the onset of an end QRS slur (defined as slurred J-wave) were designated as J_p_ and exceeded 0.1 mV in inferior and/or lateral leads of a standard 12-lead ECG at admission [19, 26]. Additionally, survival status at 28 days was recorded. If patients were discharged from the hospital within this period, follow-up was conducted with the help of the distant data approach “ProMed” (Program for Medical Cases Monitoring) and patients were contacted by phone to assess survival status. In all survivors presenting with J-waves on admission, if available, a follow-up ECG at six to eight months was analyzed for persistence of J-waves. Follow-up ECGs were either performed directly in the clinic or by the primary care physician with subsequent transmission to our clinic, depending on the patient’s preference. In total, 15 patients were lost to follow-up in the J-wave cohort (Fig 1).

### Statistical analyses

The statistical analysis was carried out by our blinded statistical analytic team using SPSS software (package 21 and R-Studio). Continuous data are presented as median and interquartile range [IQR] values and variables were compared using the Mann-Whitney-U test as statistical criteria for determining differences in the groups as having the greatest statistical power among non-parametric tests with small sample sizes. Categorical variables are reported as percentages and were compared using the Fisher-exact test. A p-value <0.05 was regarded as statistically significant. Univariate Cox survival models and multivariable Cox regression models for 28-day mortality were calculated. Results are reported as odds ratio (OR) and corresponding 95% confidence intervals (CIs).

Univariate Cox survival models were calculated to determine association of parameters with 28-day mortality rate after admission. Risk function *h*(*t* | *x*) for univariate Cox model was calculated according to the formula:

$$h(t|x)=exp(x\beta )h_{0}\left(t\right)$$ (1)

where *β* coefficient of regression of predictive variable, *x* mortality risk factor, and *h*_0_(*t*) base risk function. To estimate the *β* coefficient, the Efron approximation function of partial likelihood.

Multivariable Cox regression models for 28-day mortality were further performed including confounders with a *p*-value <0.150 in the univariate analyses. The results were reported with hazard ratios and 95% CIs. For multivariate survival model the Gsslasso Cox method was used (Bayesian hierarchical model) [27]. To estimate *β*_*j*_ coefficients for the *j*-risk predictor in the model, we used Bayesian hierarchical modeling using a double exponential prior distribution over the coefficients *β*_*j*_. The statistical significance of Cox models was assessed based on Likelihood Ratio (LR) test. To assess the quality, the measure of randomness explained (MER) was used according to the following formula:

$$R_{mer}^{2}=1-exp\left(\frac{2}{n}L_{0}-L_{1}\right)$$ (2)

where *L*_0_ and *L*_1_—represent the likelihood function for full and restricted models, and *n*—the number of deaths. The interpretation of the simulation results was carried out on the basis of an assessment for the risk ratio for each *i*-th predictor:

$$HR(x_{i})=\frac{h(t|x_{i})}{h_{0}(t)}=\text{exp}\left(x_{i}\beta \right)$$ (3)

Results of the multivariate survival Gsslasso Cox models are reported as odds ratio (OR) and corresponding 95% confidence intervals (CIs). The exp (β) multiplicator was calculated to assess the impact of each predictor.

The Kaplan-Meier method was used to generate mortality curves for descriptive purposes with censoring performed at the date of death. The Cox’s F-Test with zero survival difference hypothesis was performed and survival rates in different time intervals within 28-days were calculated.

## Results

Baseline characteristics and laboratory parameters at admission are presented in Table 1. Clinical outcomes and ECG characteristics at admission are presented in S1 Table. The mean age of our study population was 59 (49; 66) years. While oxygen saturation at admission was normal in the majority of the patients, 47.2% required oxygen therapy, 7.3% non-invasive and 5.2% invasive ventilatory therapy during hospitalization. The fatality rate was 5.2% (S1 Table).

**Table 1:** Demographics, clinical characteristics, and laboratory parameters at admission of patients hospitalized for COVID-19.

Parameter	Median (Q1; Q3) or %
N	386
Gender, m/f	40.16% / 59.84%
Age, years	59 (49; 66)
BMI, kg/m^2^	27.5 (25.03; 31.14)
***COVID-19 related symptoms at admission***:	
Dyspnea, % (n)	62.7 (238)
Cough, % (n)	10.1 (328)
Fever, % (n)	85.0 (284)
Chest pain, % (n)	21.2 (82)
Myalgia, % (n)	51.0 (197)
Dizziness, % (n)	17.4 (67)
Nausea / vomiting, % (n)	2.8 (11)
Diarrhea, % (n)	3.1 (12)
***Clinical presentation at admission***	
SpO_2_, %	97 (95; 99)
Temperature at admission, °C	36.7 (36.3; 37.3)
SAP, mm Hg	130 (120; 148)
DAP, mm Hg	85 (79; 90)
HR, beats / min	90 (78; 100)
BR, min	19 (19; 19)
Lung tissue damage on CT, %	48 (32.75; 56)
***Relevant concomittant disease***:	
AH, % (n)	48.2 (186)
DM, % (n)	10.1 (39)
CKD, % (n)	0.5 (2)
CHD, % (n)	3.6 (14)
CHF, % (n)	4.9 (19)
History of Stroke, % (n)	1.0 (4)
Obstructive lung disease, % (n)	2.3 (9)
History of AF, % (n)	3.4 (13)
Permanent, AF, % (n)	2.6 (10)
Persistent/Paroxysmal AF, % (n)	0.8 (3)
***Laboratory parameters***	
Hb, dg/l	13.0 (120; 140)
WBC, *10^9^	5,0 (3.69; 6.7)
Platelets, *10^9^	199.5 (159.25; 259.75)
ESR, mm/sec	29 (18; 42)
CRP, mmol/l	23.4 (10; 57.8)
Procalcitonin, U	0.1 (0.04; 0.14)
Albumin, g/l	40.3 (37.7; 42.9)
CK, n (%)	106.5 (65; 210.75)
Urea, mmol/l	5.1 (4.19; 6.45)
GFR, ml/min/m^2^	91.9 (72.58; 107.26)
D-Dimer, ng/ml	270 (150; 350)
Sodium, mmol/l	142 (140; 144)
Potassium, mmol/l	4.2 (3.9; 4.5)

AH–arterial hypertension, BA–bronchial asthma, CK–creatine kinase, CHD–coronary heart disease, CHF-congestive heart failure, CKD–chronic kidney disease, CRP- C-reactive protein, CT computer tomography, DBP–diastolic blood pressure, DM–Diabetes Mellitus type 2, ESR–erythrocytes sedimentation rate, Hb–hemoglobin, HR–heart rate, MI–myocardial infarction, SBP–systolic blood pressure, WBC–white blood count.

Results of the ECG analyses are depicted in S1 Table and Table 2. Repolarization abnormalities were a frequent finding in admission ECGs predominantly T wave inversions (10.6%; S1 Table). Additionally, we also observed a frequent downward R-wave serration consistent with J-waves [17–19]. The incidence of J-waves was 12.2% in our study population (n = 47/386). Consistent with previous studies [13], we observed two J-wave morphologies (notched 31.2%, slurred 40.4, both notched and slurred 28.4%), in the inferior and lateral leads (Table 2). Of note, these changes also appeared in the absence of further repolarization abnormalities (Table 2).

**Table 2:** Distribution of J-waves and other repolarization abnormalities on ECG leads.

Lead/ ECG features	I	II	III	aVF	aVL	V1	V2	V3	V4	V5	V6
J wave, % (n)	46.8 (22)	21.2 (10)	51.0 (24)	51.0 (24)	_	_	_	_	_	12.7 (6)	48.9 (23)
T-wave inversion, % (n)	4.2 (2)	21.2 (10)	21.2 (10)	8.5 (4)	14.9 (7)	10.6 (5)	8.5 (4)	10.6 (5)	8.5 (4)	8.5 (4)	8.5 (4)
ST elevation, % (n)	_	2.1 (1)	2.1 (1)	_	_	4.2 (2)	12.8 (6)	12.8 (6)	4.2 (2)	2.1 (1)	_
ST depression, % (n)	2.1 (1)	2.1 (1)	2.1 (1)	_	_	_	2.1 (1)	4.2 (2)	4.2 (2)	4.2 (2)	_

We further characterized the patients with and without J-waves and conducted a comparison between the two groups. The findings are presented in Tables 3 and 4.

**Table 3:** Demographic and clinical characteristics of COVID-19 patients with J-waves versus non-J-waves.

Parameter	J-waves	Non-J-waves	p
n	47	339	
Gender, m/f, (%)	25.5/74.5	42.5/57.5	0.027*
Age, years	62 (58; 72.5)	58 (48.5; 66)	<0.001*
BMI, kg/m^2^	29.6 (26.6; 32.6)	27 (24.8; 31)	0.003*
***COVID-19 related symptoms at admission***:			
Dyspnea, % (n)	72.3 (34)	60.2 (204)	0.108
Cough, % (n)	80.9 (38)	85.6 (290)	0.399
Fever, % (n)	61.2 (29)	75.2 (255)	0.049*
Chest pain, % (n)	27.7 (13)	20.4 (69)	0.251
Myalgia, % (n)	38.3 (18)	52.8 (179)	0.063
Dizziness, % (n)	10.6 (5)	18.3 (62)	0.194
Nausea/vomiting, % (n)	0 (0)	3.2 (11)	0.210
Diarrhea, % (n)	2.1 (1)	3.2 (11)	0.679
***Clinical presentation at admission***:			
SpO_2_, %	97 (95; 98)	97 (95; 99)	0.370
Temperature at admission, °C	36.6 (36.2; 37)	36.7 (36.3; 37.3)	0.300
SAP, mm Hg	134 (122; 150)	130 (120; 146)	0.36
DAP, mm Hg	85 (80; 90)	85 (79; 90)	0.740
HR, beats/min	94 (78.5; 100)	90 (78; 100)	0.630
BR, min	19 (18; 19)	19 (19; 19)	0.075
Lung tissue damage, %	48 (40; 58)	46.5 (32; 56)	0.136
***Relevant concomitant diseases***:			
AH, % (n)	55.3 (26)	47.8(162)	0.296
DM, % (n)	14.9 (7)	9.4 (32)	0.245
CKD, % (n)	2.1 (1)	0.3 (1)	0.101
CHD, % (n)	2.1 (1)	3.8 (13)	0.557
CHF, % (n)	14.9 (7)	3.5 (12)	<0.001*
History of MI, % (n)	2.1 (1)	2.9 (10)	0.751
History of Stroke, % (n)	4.2 (2)	0.6 (2)	0.034*
Obstructive lung disease, % (n)	4.2 (2)	2.0 (7)	0.281
History of atrial fibrillation:	6.4 (3)	2.9 (10)	0.222
Permanent, % (n)	6.4 (3)	2.6 (9)	0.171
Persistent/Paroxysmal, % (n)	0 (0)	1 (0.3)	-
***Laboratory parameters***			
Hb, g/l	13.0 (122; 142)	13.0 (120; 139)	0.310
WBC, *109	6.1 (4.51; 8.17)	4.8 (3.64; 6.5)	0.005*
Platelets, *109	190 (146; 263)	201 (160; 260)	0.310
ESR, mm/sec	31 (22; 46)	28 (18; 42)	0.271
CRP, mmol/l	30.6 (14.6; 66)	22.5 (10; 56,7)	0.112
Procalcitonin, U	0.06 (0.02; 0.18)	0.1 (0.04; 0.14)	0.973
Albumin, g/l	40.4 (37.4; 42.7)	40.3 (37.8; 42.9)	0.849
CK, % (n)	100 (58; 213)	107 (66; 210)	0.638
Urea, mmol/l	5.6 (4.8; 7.6)	5.1 (4.1; 6.3)	0.289
GFR, ml/min/m2	79.5 (57.2;103.4)	92.7 (74.6; 108.5)	0.443
D-Dimer, ng/ml	270 (150; 374)	270 (150; 343)	0.936
AST, mmol/l	27 (22.3; 43.4)	28.2 (21.6; 39.3)	0.936
ALT, mmol/l	25.2 (17.6; 39)	26.5 (17.7; 45.5)	0.634
Potassium, mmol/l	4.3 (3.95; 4.6)	4.2 (3.9; 4.5)	0.229
Sodium, mmol/l	141.0 (139.4; 144)	142.0 (140; 144)	0.246

AH–arterial hypertension, BA–bronchial asthma, CK–creatine kinase, CHD–coronary heart disease, CHF-congestive heart failure, CKD–chronic kidney disease, CRP- C-reactive protein, CT computer tomography, DBP–diastolic blood pressure, DM–Diabetes Mellitus type 2, ESR–erythrocytes sedimentation rate, Hb–hemoglobin, HR–heart rate, MI–myocardial infarction, SBP–systolic blood pressure, WBC–white blood count.

*p<0.05.

**Table 4:** ECG characteristics and clinical outcomes in COVID-19 patients with J-waves versus non-J-waves.

Parameter	J-waves	Non- J-waves	P
n	47	339	
***ECG characteristics***			
QRS duration, msec	100 (80; 100)	60 (60; 80)	0.005*
Corrected QT (Bazett), msec	426 (393; 457)	385 (362; 413)	<0.001*
T wave inversion, % (n)	38.3 (18)	5.3 (18)	<0.001*
ST elevation, % (n)	14.9 (7)	0.6 (2)	0.161
ST depression, % (n)	10.6 (5)	0.3 (1)	0.223
***Relevant outcomes***:			
Need for oxygen support, % (n)	34.0 (16)	49.0 (166)	0.055
Need for non-invasive ventilation, % (n)	12.8 (6)	6.5 (22)	0.120
Need for invasive ventilation, % (n)	8.5 (4)	4.7 (16)	0.272
Ventilated, days	6 (4; 7)	4 (3; 6)	0.098
Hospital stay, days	11 (10; 13.5)	11 (10; 14)	0.720
28-days mortality, % (n)	14.9 (7)	3.8 (13)	0.001*

*p<0.05.

In our study population, J-waves at admission were more common in older and female patients as well as in patients with a higher BMI. Furthermore, history of congestive heart failure and/or stroke were more often observed in this patient collective. There was no difference with regard to body temperature between the J-wave and the non-J-wave cohorts, while white blood count was more elevated in patients with J-waves (Table 3). Apart from J-waves on the admission ECG, COVID-19 patients presented with longer QRS and QTc intervals as well as with higher incidence of T-wave inversion.

With regard to outcomes, the 28-day mortality rate was significantly higher in the J-wave cohort (J-wave: 14.9% vs. non-J-waves 3.8%, p = 0.001, (Table 4)). Furthermore, in the univariate Cox survival model, incidence of J-waves was also linked to 28-day mortality (LR 6.09, p = 0.014, S2 Table). Further variables associated with the 28-day mortality rate using the univariate survival Cox regression model (p<0.15) included: age, chronic kidney disease, history of stroke, coronary heart disease, arterial hypertension, obstructive lung disease, history of atrial fibrillation, GFR, Hb, albumin as well as the ECG parameter ST-elevation on admission ECG (S2 Table).

**Fig 2:**
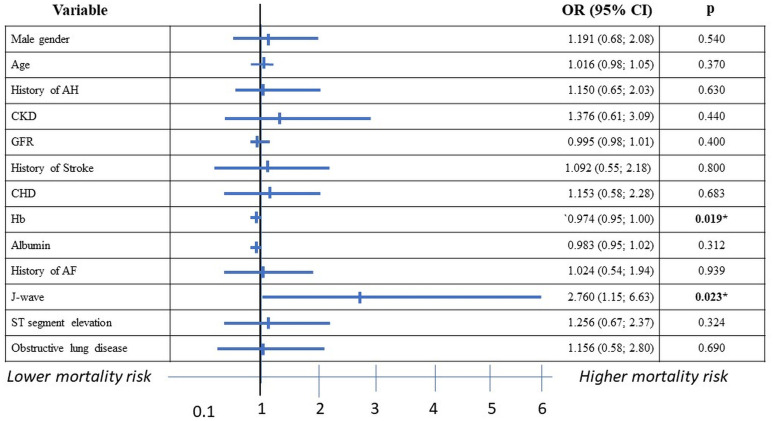
Independent predictors of 28-day mortality from COVID-19 in multivariable logistic-regression analysis. Results are reported as odds ratios (OR) and 95% confidence intervals (CIs). AF–atrial fibrillation, AH–arterial hypertension, CHD–coronary heart diseases, CKD–chronic kidney disease, Hb–hemoglobin. *p<0.050.

**Fig 3:**
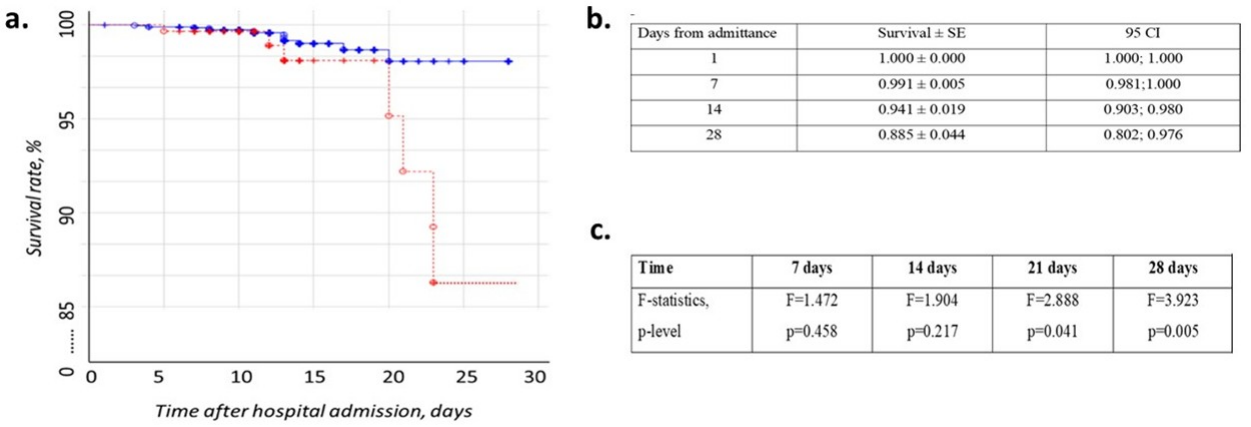
a) Kaplan-Maier survival curves of patients with COVID-19 with (blue) and without (red) J-waves within 28 days. b) Kaplan-Maier multiplier survival values within 28 days from hospitalization in patients with COVID-19. c) Survival rates difference between J- and no J-wave patients.

To further, verify an independent association of J-waves with 28-day mortality, we performed multivariable Gsslasso Cox analyses using the variables described above as well as the confounder gender. The Likelihood ratio of the applied model was 24.0 (p<0.001), R_mer^2 = 0.61 (Fig 2). Of note, in addition to Hb levels at admission and the incidence of J-waves remained the only indicator associated with fatal events. Among the presented variables, J-waves had the largest impact on survival (OR 2.76 95% CI: 1.15–6.63; p = 0.023, Fig 2), indicating J-waves on admission ECG to be predictive for mortality in COVID-19. To elucidate differences in survival between the two groups, multiple Kaplan-Meier analyses were further applied. Calculated values for the periods 7, 14, 21 and 28 days are summarized in Fig 3. According to Cox’s F-Test results with zero survival difference hypothesis, incidence of J-waves decreased COVID-19 survival rate from day 21 of admittance (Fig 3).

**Fig 4:**
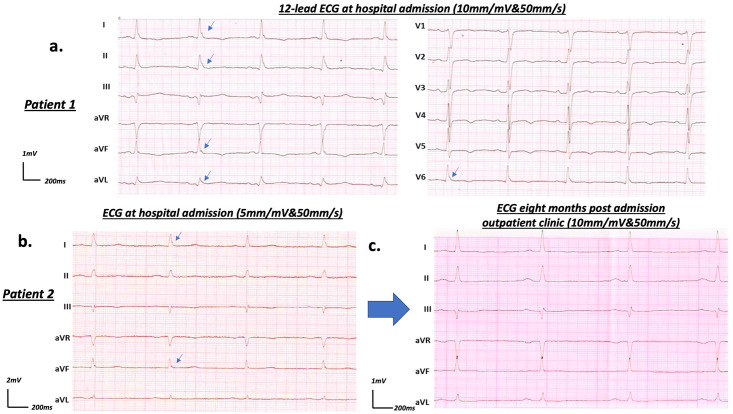
a) Admission ECG (10mm/mV&50mm/s) of a 74-year-old female (patient 1) with COVID-19 pneumonia presenting J-waves (blue arrow). During hospitalization, the patient developed respiratory failure, required mechanical ventilation and died on the 20^th^ day upon admission. b-c) Limb leads recording from 12-lead ECGs of a 72-year-old male (patient 2) admitted to hospital with COVID-19 pneumonia b) At admission J-waves were revealed (blue arrow) on the limb recordings (5mm/mV&50mm/s) but the patients could be successfully discharged on 13^th^ day upon hospitalization. c.) During follow-up at eight months, ECG (10mm/mV&50mm/s) performed in an outpatient clinic revealed a resolution of J-waves.

In addition to these findings, we further aimed to evaluate if J-waves might be a transient finding and therefore specifically related to acute COVID-19 disease. Accordingly, we analyzed available follow-up ECGs obtained at six to eight months after hospital discharge in those patients presenting with J-waves at admission. We were able to obtain follow-up ECGs in 22 patients in this cohort (Fig 4). We noted resolution or significant decline of J-waves in eight (36.4%) of the studied patients (Fig 4b), indicating that J-waves are partially transient in acute COVID-19 disease.

## Discussion

J-waves are an ECG feature observed in 5–6% of the general population according to previous studies [17, 18]. Although the condition is usually considered benign, it is associated with an increased risk of cardiovascular death, predominantly triggered by ventricular arrhythmias [17, 18]. The frequent finding of J-waves in patients hospitalized for COVID-19 in the course of the pandemic attracted the attention of authors of this manuscript. Accordingly, we hypothesized that J-waves on admission might be linked to disease-specific pathophysiologic processes and might also impact prognosis. Indeed, 12.2% of our patient collective presented with J-waves in the admission ECG, which revealed typical morphology and localization characteristics [17, 18]. However, the incidence of this phenomenon in our study collective was higher (12.2%) compared to the general population (5–6%) [17, 18]. The presence of J-waves is associated with two inherited arrhythmogenic disorders: Brugada-Syndrome and Early Repolarization Syndrome (ERS). In our study cohort, no patient fulfilled the ECG-criteria for the diagnosis of Brugada syndrome. Additionally, QTc-intervals were within normal range, a finding which suggests ERS is not a potential confounder. The finding of normal QTc-intervals is of further significance as hypercalcemia is typically associated with a short QTc-interval [19]. While we did not measure calcium levels, the finding of normal QTc-intervals disproves hypercalcemia as a relevant confounder of our findings. Hypothermia was reported as a further trigger for J-waves [19]. However, all of our patients were normothermic. Moreover, there was no difference in body temperature at admission between the J-wave and the non-J-wave cohort (Table 3). Nevertheless, the presence of fever was less frequent in the J-wave cohort. This is in line with previous basic research reports which described a temperature dependency of this electrophysiological feature [28]. Furthermore, a potential association of J-waves with different psychotropic drugs especially in the context of hypothermia was reported [29]. Additionally, onset of J-waves in response to propofol has been observed [30]. Moreover, another study discussed the attenuation of J-waves in response to medical treatment with quinidine as potential indicator of therapy efficacy in ERS [31, 32]. These findings have to be considered, when interpreting our results. However, no psychiatric disorders were documented in the baseline characteristics of our study cohort. Additionally, propofol and/or quinidine were not administered to our patient population at admission (S3 Table). Thus, a relevant impact of drug therapy on the incidence of J-waves in our study collective seems unlikely.

Interestingly, J-wave-syndrome was previously associated with male sex according to earlier studies [33]. Contrary to the reported predominance of J-waves in male patients, in our study we observed a female predominance with 75% of patients in the J-wave cohort being women. However, it must be emphasized that J-wave patients in our study did not meet the Brugada or ERS criteria. Still, we cannot rule out a potential impact of COVID-19 on hormonal homeostasis as a potential explanation for this finding. On the other hand, male patients had a higher mortality rate, thus potentially accounting for a selection bias. However, given the comparably small sample size, the sex-differences observed in our study might also be attributable to chance.

The J-wave specification (and its amplitude) are based on a transmural potassium gradient, promoted by increased I_to_-potassium current activity in the epicardium [28]. While I_to_ activity is also upregulated during hypoxic events through K_ATP_-channel activation, [34] J-waves are associated with ischemic but also non-ischemic cardiac damage [35, 36]. In our cohort, J-waves were present more frequently in patients with heart failure and older age (Table 3), conditions which both promote cardiac injury. However, history of CHD was not significantly different between the J-wave and non-J-wave cohorts. Accordingly, these factors point towards a COVID-19-related effect as potential explanation for our findings. As mentioned above, cardiac injury is a frequent finding, especially in severe COVID-19, which promotes unspecific ECG-abnormalities [9–11]. Of note, the pathophysiology behind the COVID-19-induced cardiac injury is still matter to debate. While COVID-19-related myocarditis was suspected in previous studies [9, 10], recent data show that cardiac damage in COVID-19 pneumonia is primarily caused by high inflammatory and thrombogenic activity [37, 38]. In the J-wave cohort, white blood counts were increased, indicative of a more pronounced inflammatory activity. This aligns with studies reporting an increase in K_ATP_-channel opening, a driving force of I_to_ function, during systematic inflammatory responses. Furthermore, while specific COVID-19 treatment was similar between groups (S3 Table), mortality was significantly increased in the J-wave cohort indicating a higher disease severity in this group. Markers of cardiac injury were not routinely investigated in our study with the exception of CK, although these levels are not considered cardiac-specific. Therefore, we are not able to evaluate a potential correlation of COVID-19-related cardiac injury and the observed J-wave incidence.

On the other hand, J-waves are linked to fatal arrhythmic events [17, 19]. One fatal event related to ventricular tachycardia was observed in the J-wave cohort (Fatal events: 1/7, 14.3%; S4 Table). This could suggest an increased susceptibility for malignant arrhythmias in this COVID-19 subpopulation. On the other hand, the remaining six COVID-19 fatalities in the J-wave cohort occurred in the absence of arrhythmic events (S4 Table). Thus, further studies need to elucidate the impact of J-waves on (fatal) arrhythmias in COVID-19.

Furthermore, J-waves resolved or declined in 36.4% of patients in which a follow-up ECG was available (22 from 47 patients). Here, one could speculate that persistence of J-waves in the remaining ECGs might be associated with a preexistence of this feature [17, 18]. This would indicate J-waves to be a transient and likely disease-specific finding. However, since preexisting ECGs were not available in this retrospective study, this speculation must be treated with caution.

While our study is not able to fully elucidate the pathophysiology of J-waves in COVID-19, to the best of our knowledge, this is the first report which examines the high incidence of this ECG feature in acute COVID-19. Importantly, J-waves on admission ECGs were independently linked to COVID-19 mortality and were revealed as the strongest predictor of case fatality. As ECG analysis constitutes an easily applicable and inexpensive clinical tool, identification of J-wave patterns represents a promising prognostic approach, also for overstrained medical systems. Identification of J-wave patterns could aid in identifying high-risk patients and thus improve clinical care during the pandemic.

### Limitations

The present study has by design its limitations, while contributing novel clinical findings. One is its single-centre and retrospective design. Among others, this might result in bias caused by applied hospital-specific standards of patient care and specific patient characteristics. Nevertheless, COVID-19 management was in accordance with Russian Federation’s National Guidelines and the Bashkortostan Republic is characterized by a broad ethnic variety. Due to an overstrained medical system caused by the pandemic, cardiac enzymes were not routinely assessed and cardiac imaging was not routinely performed which would help to further characterize cardiac involvement in our cohort. This limitation also applies for additional inflammatory markers such as interleukine-6. Also, no serum calcium measurements were done which may show hypercalcemia as a reason for J-wave appearance. In hospital follow-up, ECGs were not routinely performed, and we were therefore not able to access out of hospital follow-up ECGs as well as prehospital ECGs in all J-wave patients. Furthermore, continuous close rhythm monitoring was not available to assess arrhythmia risk in our cohort. The compassionate use of untested treatments in a number of COVID-19 patients (S3 Table) might have affected the results. Moreover, it is important to emphasize that our findings only account for hospitalized patients with potentially more severe infection. Accordingly, our findings might not be applicable in patients with mild disease not requiring hospitalization. Another important point is that J-wave occurrence might be only a transient finding. Thus, Holter-ECG monitoring would have been necessary for the detection of transient or dynamic J-wave appearance in our study cohort [39].

### Conclusion and perspective

In conclusion, according to our results, we propose J-wave patterns in the admission ECG as a potential prognostic factor with regards to 28-day mortality in COVID-19 patients requiring hospitalization. The application of our findings could help to identify patients at increased risk and consequently to improve clinical care during the pandemic. Still, the pathophysiologic background as well as the question of a COVID-19 specificity of our findings need further elucidation.

### Future work

Based on our findings, we propose a further investigation of J-waves in COVID-19 patients especially with respect to a potential correlation with myocardial damage and other frequent cardiovascular pathologies. Additionally, also the long-term prognostic impact of J-waves in the admission ECGs of COVID-19 patients needs further clarification. Moreover, the evaluation of the pathophysiologic processes involved in the development of J-waves in patients more severely affected by COVID-19 could further contribute to the extension of knowledge on the cardiovascular impact of COVID-19 itself.

## Supporting information

S1 Table Relevant clinical outcomes and relevant ECG characteristics in COVID-19 upon admission. (DOCX)

S2 Table Univariate Cox regression for risk factors of mortality in COVID-19 patients with p<0.150. (DOCX)

S3 Table Medication of COVID-19 patients. (DOCX)

S4 Table Characteristics of deceived patients with J-wave. (DOCX)

S1 File (RAR)

## Abbreviations

AH: arterial hypertension
BA: bronchial asthma
CHD: coronary heart disease
CHF: congestive heart failure
CK: creatine kinase
CKD: chronic kidney disease
COVID-19: COronaVIrus Disease 2019
CRP: C-reactive protein
CT: computer tomography
DBP: diastolic blood pressure
DM: Diabetes Mellitus type 2
ECG: electrocardiography
ESR: erythrocytes sedimentation rate
Hb: hemoglobin
HR: heart rate
MI: myocardial infarction
SBP: systolic blood pressure
WBC: white blood count
