# Supplementary material for: J-waves in acute COVID-19: A novel disease characteristic and predictor of mortality?
Source: PLoS One. 2021 Oct 14;16(10):e0257982. doi: 10.1371/journal.pone.0257982 (PMC8516278; doi:10.1371/journal.pone.0257982)
Supplement: S1 Table — (DOCX) [file pone.0257982.s001.docx]

**S1 Table. Relevant clinical outcomes and relevant ECG characteristics in COVID-19 upon admission**

| **Parameter** | **Median (Q1; Q3) or %** |
| --- | --- |
| N | 386 |
| ***ECG characteristics***  QRS duration, msec  Corrected QT (Bazett), msec  T wave inversion, % (n)  ST elevation, % (n)  ST depression, % (n)  J-waves, % (n)  Notched J-waves, % (n)  Slurred J-waves, % (n)  Notched and slurred J-waves, % (n) | 60 (60; 80)  388.2 (363.65; 416.77)  10.6 (41)  4 (14)  2 (8)  12.2 (47)  31.2 (15)  40.4 (19)  28.4 (13) |
| ***Relevant outcomes:***  Need for oxygen support, % (n)  Need for non-invasive ventilation, % (n)  Need for invasive ventilation, % (n)  Ventilated, days  Hospital stay, days  28 days mortality, % (n) | 182 (47.2)  28 (7.3)  30 (5.2)  5 (3; 6)  11 (10;14)  20 (5.2) |

T wave inversion, ST elevation or ST depression was diagnosed, if presented on at least one ECG lead on a 12-lead ECG at admission.
