# Supplementary material for: J-waves in acute COVID-19: A novel disease characteristic and predictor of mortality?
Source: PLoS One. 2021 Oct 14;16(10):e0257982. doi: 10.1371/journal.pone.0257982 (PMC8516278; doi:10.1371/journal.pone.0257982)
Supplement: S2 Table — (DOCX) [file pone.0257982.s002.docx]

**S2 Table. Univariate Cox regression for risk factors of mortality in COVID-19 patients with p<0.150**

| **Risk factor** | **Coefficient ± SE** | **p-Value** | **Likehood Ratio (LR)** | **p-value of LR** |
| --- | --- | --- | --- | --- |
| Male gender | 0.044 | 0.728 | 8.51 | 0.006* |
| Age | 0.017 | 0.008 | 8.51 | 0.006* |
| CKD | 58.877 | <0.001 | 11.84 | <0.001* |
| GFR | 0.978 | 0.003 | 8.7 | 0.003* |
| History of stroke | 9.330 | 0.032 | 2.62 | 0.106 |
| CHD | 3.569 | 0.091 | 2.08 | 0.149 |
| Hb | 0.966 | < 0.001 | 9.72 | 0.002* |
| Albumin | 0.952 | 0.015 | 4.65 | 0.031* |
| History of AF | 3.776 | 0.035 | 3.27 | 0.071* |
| J-waves | 3.535 | 0.007 | 6.09 | 0.014* |
| ST elevation | 2.714 | 0.032 | 3.95 | 0.047* |
| History of AH | 2.231 | 0.610 | 3.79 | 0.051* |
| Post MI | 3.574 | 0.092 | 2.08 | 0.149 |
| RBC | 0.308 | <0.001 | 11.13 | <0.001* |
| Creatinine | 1.008 | 0.029 | 3.02 | 0.082 |
| Obstructive lung disease | 3.599 | 0.087 | 2.12 | 0.145* |
| Height | 0.985 | 0.524 | 0.41 | 0.522 |
| Weight | 0.980 | 0.216 | 1.62 | 0.203 |
| BMI | 0.972 | 0.479 | 0.52 | 0.472 |
| BR | 1.005 | 0.968 | 0 | 0.969 |
| SpO_2_ | 0.984 | 0.316 | 0.70 | 0.403 |
| Temperature | 0.856 | 0.542 | 0.38 | 0.535 |
| SBP | 0.994 | 0.544 | 0.36 | 0.551 |
| DBP | 0.989 | 0.497 | 0.44 | 0.505 |
| HR | 0.993 | 0.662 | 0.19 | 0.662 |
| DM | 0.555 | 0.335 | 1.00 | 0.316 |
| CHF | 2.501 | 0.147 | 1.7 | 0.192 |
| COVID-19 lung damage | 0.992 | 0.46 | 0.55 | 0.460 |
| Procalcitonin | 1.000 | 0.963 | 1 | 0.963 |
| D-Dimer | 1.000 | 0.486 | 0.4 | 0.529 |
| PLT | 0.996 | 0.175 | 2.01 | 0.156 |
| ESR | 1.004 | 0.728 | 0.12 | 0.729 |
| WBC | 1.010 | 0.569 | 0.27 | 0.603 |
| CRP | 1.002 | 0.746 | 0.1 | 0.747 |
| AST | 1.003 | 0.353 | 0.65 | 0.421 |
| ALT | 1.001 | 0.483 | 0.32 | 0.570 |
| Sodium | 0.978 | 0.383 | 1.27 | 0.295 |
| Potassium | 0.723 | 0.43 | 0.65 | 0.400 |
| CK | 0.999 | 0.211 | 3.23 | 0.722 |
| QRS | 6250.53 | 0.395 | 0.7 | 0.403 |
| QT(cor) | 0.999 | 0.824 | 0.05 | 0.824 |
| ST depression | 1.231 | 0.84 | 0.04 | 0.845 |
| Negative T wave | 2.160 | 0.224 | 1.24 | 0.265 |

AH–arterial hypertension, BA–bronchial asthma, CK–creatine kinase, CHD–coronary heart disease, CHF-congestive heart failure, CKD–chronic kidney disease, CRP- C-reactive protein, CT computer tomography, DBP–diastolic blood pressure, DM–Diabetes Mellitus type 2, ESR–erythrocytes sedimentation rate, Hb–hemoglobin, HR–heart rate, MI–myocardial infarction, SBP–systolic blood pressure, WBC–white blood count. *p<0.150
