# Supplementary material for: J-waves in acute COVID-19: A novel disease characteristic and predictor of mortality?
Source: PLoS One. 2021 Oct 14;16(10):e0257982. doi: 10.1371/journal.pone.0257982 (PMC8516278; doi:10.1371/journal.pone.0257982)
Supplement: S3 Table — (DOCX) [file pone.0257982.s003.docx]

**S3 Table. Medication of COVID-19 patients**

| ***Parameter*** | All | J-waves | Non-J-waves | p |
| --- | --- | --- | --- | --- |
| N | 386 | 47 | 339 |  |
| ***Relevant cardiovascular medication*** |  |  |  |  |
| ACE/ARA, % (n) | 20.0 (81) | 23.4 (11) | 20.1 (68) | 0.595 |
| Diuretics, % (n) | 4.9 (19) | 2.1 (1) | 4.7 (18) | 0.345 |
| Aldosterone antagonist, % (n) | 9.8 (38) | 6.3 (3) | 9.1 (35) | 0.396 |
| Ca^2+^-channel blocker, % (n) | 11.0 (46) | 12.8 (6) | 11.8 (40) | 0.849 |
| Beta-blocker, % (n) | 20.4 (79) | 23.4 (11) | 20.6 (70) | 0.694 |
| Class I and III antiarrhythmics, % (n) | 0 (0) | 0 (0) | 0 (0) | >0.999 |
| ***COVID-19 related medication*** |  |  |  |  |
| Hydroxychloroquine, % (n) | 58.2 (225) | 53.2 (25) | 59.0 (200) | 0.449 |
| Lopinavir and Ritonavir, % (n) | 36.0 (139) | 36.2 (17) | 31.6 (122) | 0.980 |
| Tozilisumab, % (n) | 8.0 (31) | 14.9 (7) | 7.1 (24) | 0.065 |
| Glucocorticosteroids, % (n) | 100 (386) | 100 (47) | 100 (339) | 0.981 |
| Therapeutic low-weight heparin, % (n) | 96.1 (371) | 97.9 (46) | 95.9 (325) | 0.506 |

ACE=angiotensin-converting-enzyme inhibitors, ARA=angiotensin receptor antagonist, Ca^2+^-channel blocker= Ca^2+^-channel blocker of dihydropyridine and nondihydropyridine type
