# Supplementary material for: J-waves in acute COVID-19: A novel disease characteristic and predictor of mortality?
Source: PLoS One. 2021 Oct 14;16(10):e0257982. doi: 10.1371/journal.pone.0257982 (PMC8516278; doi:10.1371/journal.pone.0257982)
Supplement: S4 Table — (DOCX) [file pone.0257982.s004.docx]

**S4 Table. Characteristics of deceived patients with J-wave**

|  | Day of death | Sex | Age  (years) | Lung damage in CT at admission, (%) | Non-invasive ventilation | Intubation | VT/VF |
| --- | --- | --- | --- | --- | --- | --- | --- |
| 1 | 13 | female | 70 | 88 | x | x | - |
| 2 | 23 | male | 61 | 80 | x | x | - |
| 3 | 20 | female | 75 | 36 | x | x | - |
| 4 | 23 | male | 57 | 80 | x | - | - |
| 5 | 5 | female | 57 | 24 | x | x | - |
| 6 | 12 | male | 72 | 10 | - | x | x |
| 7 | 21 | female | 88 | 44 | x | - | - |

CT – computer tomography, VT – ventricular tachycardia, VF – ventricular fibrillation
